# The Level of Serum Albumin Is Associated with Renal Prognosis in Patients with Diabetic Nephropathy

**DOI:** 10.1155/2019/7825804

**Published:** 2019-02-17

**Authors:** Junlin Zhang, Rui Zhang, Yiting Wang, Hanyu Li, Qianqian Han, Yucheng Wu, Tingli Wang, Fang Liu

**Affiliations:** Division of Nephrology, West China Hospital of Sichuan University, Chengdu 610041, China

## Abstract

**Objective:**

Although hypoalbuminemia is frequently found in most patients with diabetic nephropathy (DN), its relationship to the severity and progression of DN remains largely unknown. Our aim was to investigate the association between the serum albumin levels and clinicopathological features and renal outcomes in patients with type 2 diabetes mellitus (T2DM) and biopsy-proven DN.

**Materials and Methods:**

A total of 188 patients with T2DM and biopsy-proven DN followed up for at least one year were enrolled. The patients were divided into four groups based on the albumin levels: normal group: ≥35 g/L (*n* = 87); mild group: 30-35 g/L (*n* = 34); moderate group: 25-30 g/L (*n* = 36); and severe group: <25 g/L (*n* = 31). The renal outcome was defined by progression to end-stage renal disease. The impact of the serum albumin level on renal survival was estimated using Cox regression analysis.

**Results:**

Among the cases, the serum albumin level had a significant correlation with proteinuria, renal function, and glomerular lesions. A multivariate Cox regression analysis indicated that the severity of hypoalbuminemia remained significantly associated with an adverse renal outcome, independent of clinical and histopathological features. In reference to the normal group, the risk of progression to ESRD increased such that the hazard ratio (HR) for the mild group was 2.09 (95% CI, 0.67-6.56, *p* = 0.205), 6.20 (95% CI, 1.95-19.76, *p* = 0.002) for the moderate group, and 7.37 (95% CI, 1.24-43.83, *p* = 0.028) for the severe group.

**Conclusions:**

These findings suggested that hypoalbuminemia was associated with a poorer renal prognosis in patients with T2DM and DN.

## 1. Introduction

Diabetic nephropathy (DN), recently also named as diabetic kidney disease (DKD), is one of the most common diabetic microvascular complications and has become the leading cause of chronic kidney diseases in the world [1, 2]. DN develops in approximately 40% of type 2 diabetic (T2D) patients [3] and nearly 20% of whom will finally progress to end-stage renal disease (ESRD) [4]. The previous surveys reported that DN accounted for roughly 16.4% [5] and more than 44% [6] of all cases of ESRD in China and in the USA, respectively. Although the renoprotective interventions have been universally implemented to improve glycemia, blood pressure, and serum lipid regulation over the last decades, the risk of ESRD and the health burden in DN patients is still increasing [7]. Searching further insight into the pathogenesis and risk factors for DN development is extremely urgent and essential to advance clinical management of DN.

DN is greatly a heterogeneous kidney disease, with variability in clinical courses, histopathological features, and different disease trajectories. The clinical characteristics of DN have traditionally been described as glomerular hyperfiltration, persistent albuminuria, hypertension, and finally progression to renal failure. And the typical histomorphology of DN displays glomerular basement membrane (GBM) thickening, mesangial matrix expansion, nodule sclerosis, and diffuse podocyte foot process effacement [3]. Although a large body of studies has established the contribution of several factors such as severity of glomerular lesions and proteinuria in the progression of DN [8–11], the number of researches about the association between the serum albumin and biopsy-proven DN was very limited.

In this study, we aimed to investigate the relationship between serum albumin levels and the baseline clinicopathological features in 188 patients with T2DM and biopsy-proven DN and to further evaluate the prognostic utility of serum albumin levels.

## 2. Materials and Methods

### 2.1. Ethical Approval

The ethics committee of West China Hospital approved this research. The study protocol was in compliance with the ethical standards laid down in the 1964 Declaration of Helsinki and its later amendments. Additional informed consent was obtained from all individual participants for whom identifying information is included in this article.

### 2.2. Patients

**Figure 1:**
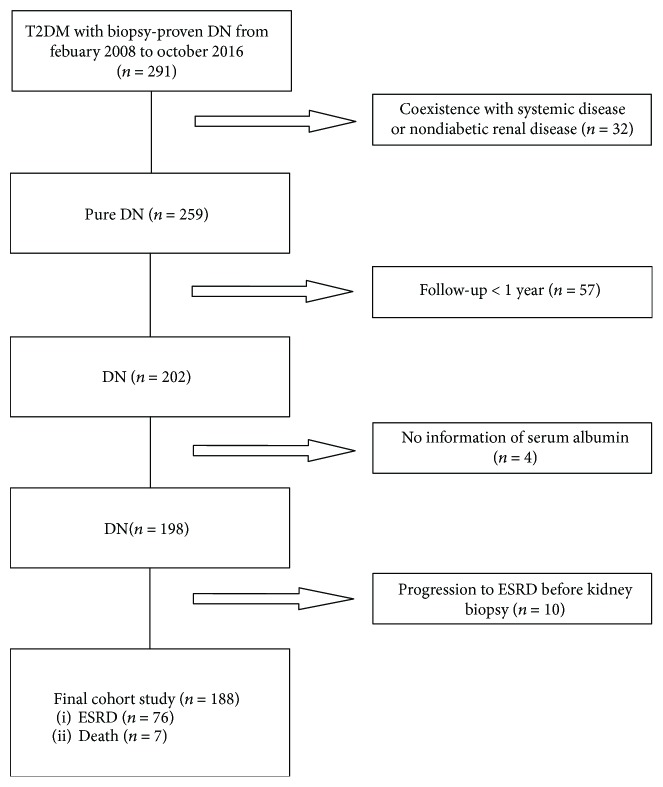
Flowchart of study participants.

A total of 291 patients with T2DM and biopsy-proven DN in West China Hospital of Sichuan University from 2008 to 2016 were reviewed, and 188 patients were eligible (Figure 1). The general indications for renal biopsy in our present study were T2DM patients with renal damage who lacked absolute contraindications, especially T2DM patients without diabetic retinopathy (DR), T2DM patients with obvious glomerular hematuria and/or sudden onset of overt proteinuria, or T2DM patients with short diabetic duration (<5 y). The diagnosis of T2DM and DN was in accordance with the standards which were established by the American Diabetes Association (ADA) in 2017 [12] and the Renal Pathology Society in 2010 [13], respectively. Exclusion criteria were the patients that coexisted with nondiabetic renal diseases (NDRDs) such as IgA nephropathy or systemic diseases, especially cancer and cirrhosis. The patients who were followed up less than 1 year, without information of the serum albumin level, or having progressed to ESRD before renal biopsy were also excluded.

### 2.3. Clinical and Pathologic Characteristics

The clinical data, including the age, gender, weight, height, history of diabetes, blood pressure, HbA1c, 24 h urinary protein, serum creatinine (mg/dL), estimated glomerular filtration rate (e-GFR, evaluated by the CKD-EPI formula), serum albumin, total cholesterol, triglyceride, and hemoglobin, were gathered at the time of renal biopsy. The information of the medication history, especially the antidiabetic agents and RAAS (renin-angiotensin-aldosterone system) inhibitor including angiotensin-converting enzyme (ACE) inhibitors or angiotensin II receptor blockers (ARBs), were also collected at baseline. The level of serum albumin was measured by the bromocresol green (BCG). Hypoalbuminemia was defined as a serum albumin level < 35 g/L, and clinically significant hypoalbuminemia was generally regarded as a level < 25 g/L [14]. Given that, the patients in this study were divided into 4 groups according to the level of serum albumin: normal albumin group (≥35 g/L), mild hypoalbuminemia group (30-35 g/L), moderate hypoalbuminemia group (25-30 g/L), and severe hypoalbuminemia group (<25 g/L). All renal biopsy specimens of this study were routinely examined by light microscopy, immunofluorescence, and electron microscopy. The histological lesions were graded according to the new criteria of the Renal Pathology Society in 2010 [13].

### 2.4. Follow-Up and Renal Outcome

The patients were followed up regularly and the information about their proteinuria and renal function was collected. The renal outcome in this study was defined by progression to ESRD, which was regarded as e − GFR < 15 mL/min/1.73 m^2^ or starting the renal replacing therapy.

### 2.5. Statistical Analysis

All statistical tests were analyzed by SPSS version 22.0 for Windows Inc. (Chicago, IL, USA). The continuous variables were presented as the mean ± standard deviation (SD) or median with IQR, and categorical data were summarized as numbers and percentages. Differences in means for quantitative variables were evaluated using ANOVA or the Kruskal–Wallis *H* test, as appropriate. Categorical variables were compared with the chi-squared test. Correlations of the serum albumin level with histopathological findings were analyzed by Spearman's correlation analysis and with the clinical findings by partial correlation adjusting for the baseline age, gender, and e-GFR. Cumulative survival was presented as Kaplan–Meier survival curves, and survival rates were assessed using the log-rank test. Cox regression models were performed to analyze the association between the levels of albumin and renal outcomes. The serum albumin level was first calculated as a continuous variable with hazard ratios (HRs) that resulted from each SD increment, and the different albumin level groups as a categorical variable, with the normal group regarded as reference. The factors that had clinical meaning or were associated with renal outcomes in univariate analysis (*p* < 0.1) were included in the multivariate Cox regression analysis. The area under the receiver operating characteristic curve (AUC) and integrated discrimination improvement (IDI) were analyzed by using logistic regression to identify the discrimination ability [15]. A two-sided *p* value <0.05 was considered statistically significant, and the Holm–Bonferroni method was applied to reduce the risk of a type 1 statistical error.

## 3. Results

### 3.1. Baseline Clinical and Pathologic Characteristics

A total of 188 patients were recruited in this study, and there is no significant difference observed between the included and excluded participants on the demographic and clinical characteristics, except for the e-GFR (Supplementary Table 1). There were 87 (46.3%) patients in the normal group, 34 (18.1%) in the mild group, 36 (19.1%) in the moderate group, and 31 (16.5%) in the severe group. At the time of renal biopsy, 129 patients were male (68.6%) and the mean age was 52.71 ± 8.79 years old. The median follow-up time was 17 (IQR, 12-103) months. The median duration of diabetes was 90 (36-132) months. The median of the serum creatinine level was 1.42 (1.02-1.82) mg/dL, the median of the e-GFR was 51.25 (37.44-77.91) mL/min/1.73 m^2^, the median of the albumin level was 34.15 (27.28-39.38) g/L, and the median of proteinuria was 4.09 (1.88-6.75) g/day at baseline. 152 (80.9%) patients received the RAAS inhibitor therapy prior to the admission. 76 patients (40.4%) progressed to ESRD from baseline during the follow-up period. The distribution by glomerular classes I, IIa, IIb, III, and IV was 5.3% (10), 18.1% (34), 10.6% (20), 50.5% (95), and 15.4% (29), respectively. The severe glomerular lesions (class III+IV) of the normal, mild, moderate, and severe groups were observed in 40 (46.0%), 27 (79.4%), 30 (83.3%), and 27 (87.1%), respectively. Clinical and pathological features of patients with different serum albumin levels are presented in Table 1.

### 3.2. Associations between the Albumin Level and the Clinicopathological Features

Among patients, we observed no significant relationship of albumin levels with the age, gender, duration of diabetes, glycosylated hemoglobin levels, and incidence of smoking and hypertension (Table 1). There was a significant trend for higher creatinine, proteinuria, and cholesterol and lower e-GFR and hemoglobin in the moderate and severe groups compared with the normal or mild groups (*p* < 0.05). The incidence of nephrotic-range proteinuria and DR was also significantly higher in the moderate and severe groups than in the other groups. There was no difference in the use of RAAS inhibitors among groups.

The correlations between the albumin levels and clinicopathological findings are illustrated in Table 2. The albumin level showed a significant inverse correlation with the glomerular class (*r* = −0.394, *p* < 0.001), as well as a weak negative relationship with the IFTA score (*r* = −0.269, *p* < 0.001), interstitial inflammation score (*r* = −0.378, *p* < 0.001), and arteriolar hyalinosis score (*r* = −0.219, *p* = 0.003). Moreover, the albumin level had a strong inverse correlation with proteinuria (*r* = −0.661, *p* < 0.001) and cholesterol (*r* = −0.424, *p* < 0.001) and a positive correlation with e-GFR (*r* = 0.334, *p* < 0.001) or hemoglobin (*r* = 0.325, *p* < 0.001) when adjusting for the baseline age, gender, and e-GFR.

### 3.3. Serum Albumin Level and Renal Outcome

**Figure 2:**
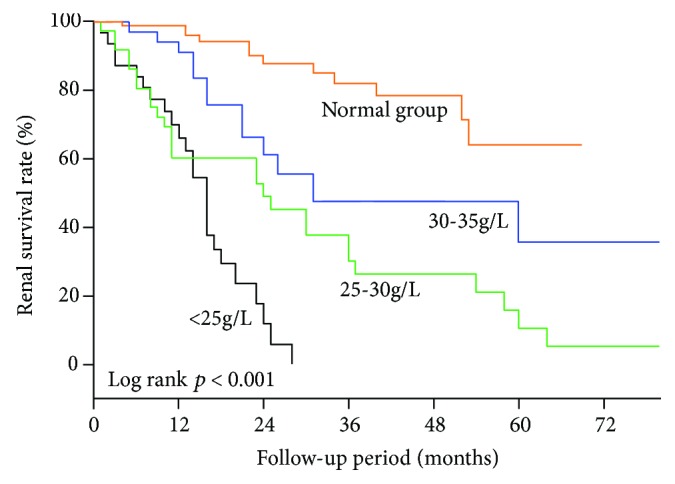
Kaplan–Meier curves of the renal survival rate in DN patients with different serum albumin levels. The 5-year renal survival rate was estimated to be 64.14%, 35.70%, 10.54%, and 0% for the normal (≥35 g/L), mild (30-35 g/L), moderate (25-30 g/L), and severe (<25 g/L) groups, respectively. Median survival time for ESRD after renal biopsy was 31 months, 24 months, and 16 months for the mild, moderate, and severe groups, respectively. There was a significant difference of the renal survival rate between any two groups (*p* < 0.05).

The Kaplan–Meier survival analysis suggested an overall 5-year renal survival rate of 29.0% in all the patients. Patients of different albumin levels had 5-year renal survival rates of 64.14% (normal group), 35.70% (mild group), 10.54% (moderate group), and 0% (severe group). Median survival time for ESRD after renal biopsy was 31 months, 24 months, and 16 months for the mild, moderate, and severe groups, respectively. As presented in Figure 2, the renal survival was significantly deteriorated by the degree of hypoalbuminemia (log-rank test, *p* < 0.001). The univariate Cox analysis demonstrated that the albumin level could significantly impact the renal outcome in these patients (hazard ratio (HR), per SD of serum albumin 0.35, 95% confidence interval (CI), 0.27-0.46, *p* < 0.001). Compared to the normal group (reference), the risk of renal failure increased by the albumin level decline: the HRs were 2.99 (95% CI, 1.36-6.61, *p* = 0.007) for the mild group, 6.03 (95% CI, 3.05-11.95, *p* < 0.001) for the moderate group, and 13.74 (95% CI, 6.63-28.44, *p* < 0.001) for the severe group. The adjusted HRs of albumin for renal outcomes are shown in Table 3. After adjusting for important clinical variables, renal pathological findings, and RAAS inhibitor use (model 3), lower levels of albumin remained independently associated with a higher risk of ESRD (HR, per SD of serum albumin 0.21, 95% CI, 0.06-0.67, *p* = 0.009). And the HRs were 2.09 (95% CI, 0.67-6.56, *p* = 0.205) for the mild group, 6.20 (95% CI, 1.95-19.76, *p* = 0.002) for the moderate group, and 7.37 (95% CI, 1.24-43.83, *p* = 0.028) for the severe group, compared with the reference. In model 3, the e-GFR was also an independent risk factor for renal prognosis in patients with DN (Supplementary Table 2).

In the multivariate model including the age, gender, e-GFR, and log (urinary protein) at baseline, addition of serum albumin as a categorical variable improved the AUC from 0.80 to 0.87. However, adding the serum albumin to the model did not improve the discrimination of the outcome (IDI = −0.16, 95% CI, (-0.21, -0.10)).

## 4. Discussion

In this study, we investigated the relationship between the serum albumin levels and clinicopathological features and renal outcomes in 188 patients with T2DM and biopsy-proven DN. The results showed that the albumin level had a significant inverse correlation with proteinuria, cholesterol, and histopathological damage, including glomerular lesions, IFTA, interstitial inflammation, and arteriolar hyalinosis, and a positive correlation with renal function and hemoglobin. Moreover, the severity of hypoalbuminemia was significantly associated with an adverse renal outcome, independent of clinical and histopathological features. Participants with the lowest level of albumin vs. the normal group demonstrated a 7.37-fold greater risk of ESRD. Thus, the results of this study suggested that low levels of serum albumin might have prognostic utility in DN. Our observations that patients with lower levels of serum albumin were more prone to progress to ESRD may alert nephrologists that such patients should be followed up closely and possibly given a more aggressive treatment.

A previous study from Japan also found that the serum albumin level was independently associated with proteinuria in Japanese DN patients [16], and another study examined the impact of hypoalbuminemia on the progression of kidney disease among 343 Caucasian (77%) and black patients with DN. They found that the hypoalbuminemia was also significantly associated with the rate of the GFR decline. However, the diagnosis of DN in their studies was not based on the pathology but on clinical manifestations. Given that nondiabetic renal disease (NDRD) is common (27-82.9%) [10, 17, 18] among diabetic patients undergoing renal biopsy, the results in their studies might be less convincing. Thus, the findings in this study performed among patients with biopsy-based diagnosis of DN may be more justified.

Human serum albumin, as the most represented plasma protein, is synthesized in the liver and secreted into the vascular space to distribute in all body tissues [14]. It plays a decisive role in the maintenance of homeostasis and makes a balance between hydrostatic and colloid osmotic pressure within vessels [19]. Serum albumin also has many physiological functions, including binding many different substances, such as hormones, ions, and drugs [20], anti-inflammatory function, and antioxidant properties [21]. Growing evidence has proven that the hypoalbuminemia was caused by an inadequate energy or protein intake, impaired liver synthesis, decreased intestinal absorption, increased tissue catabolism, or increased loss [22] and is an important risk factor and predictor of increased morbidity/mortality despite of the implicated diseases [22]. In this study, the results strongly suggested that the serum levels of albumin related to the renal prognosis. Moreover, the serum albumin level had a significant inverse correlation with proteinuria and glomerular lesions, two well-known risk factors for DN progression, which might give some explanation for the association between the hypoalbuminemia and renal outcome. However, this was by no means the only possible cause.

Moshage et al. [23] reported that albumin was a negative acute-phase protein during inflammation, and the decrease in the albumin level was associated with the effect of IL-1, IL-6, and TNF-*α* on the hepatic synthesis. Zhang and Frei [24] found that normal concentrations of albumin could selectively inhibit TNF-*α*-induced expression of vascular cell adhesion molecule-1 (VCAM-1) to attenuate the inflammation. And the results of Michelis et al. [25–27] indicated that the modified/oxidized albumin molecules in diabetic patients have a key role in accelerating oxidative stress (OS), inflammation, and endothelial injury involving neutrophil activation due to possessing the proinflammatory properties. So, the level of serum albumin, to some extent, could reflect the degree of inflammation and OS. Taken all the above contributors together, it was possible that the chronic inflammatory state [28], reflected as hypoalbuminemia, accelerated the process of kidney function deterioration, possibly by inducing oxidative stress [25, 26, 29] and endothelial inflammatory injury [27]. That hypothesis has also been evidenced in both the nondiabetic [30, 31] and diabetic patients [32].

Generally, the patients with DN were often accompanied by a disorder of lipid metabolism. Unfortunately, in our study, the hypoalbuminemia was negatively correlated with the level of cholesterol, which in turn aggravated the dyslipidemia, causing a vicious circle that leads to more severe toxic injury to the kidney induced by the lipid. In addition, this study also found that the serum level of albumin positively correlated with the level of e-GFR and hemoglobin. The patients with a lower albumin level had a decreased renal function and hemoglobin level. Growing evidence suggested that impaired kidney function might lead to accumulation of inflammatory factors, which could, in return, aggravate kidney injury [33]. Moreover, the progression of DN could further bring about decreased protein and energy intake, and, consequently, malnutrition with a resultant more severe hypoalbuminemia [34]. Moreover, the patients with hypoalbuminemia were also prone to have anemia, which might result in an anemia-induced hypoxia that accelerates kidney injury in these patients [35]. Nevertheless, the exact underlying mechanism of the hypoalbuminemia associated with the kidney prognosis needs more clinical and experimental evidence to verify.

It is noteworthy that the results of this study indicated the prognostic impact of hypoalbuminemia; the subsequent question was how to improve the renal prognosis of these patients. Growing evidence suggested that the treatment with RAAS inhibitors was effective not only to reduce the level of albuminuria but also to maintain the level of serum albumin [36]. Moreover, several experimental and clinical studies reported that pentoxifylline can retard kidney disease progression and reduce the albuminuria level due to possessing the antioxidant, anti-inflammatory, and antifibrotic properties [37–40]. Beraprost sodium (BPS), a prostaglandin analogue, also had the capacity to improve renal function and decrease urinary protein excretion via reducing inflammatory cytokine production and oxidative stress, evidenced in DN rats [41]. However, whether the latent benefits of the anti-inflammatory and antiproteinuric effect, to some extent, relied on an increase in the serum albumin level was unknown in these setting. In addition, a low-protein diet (LPD) has been advised to improve uremic symptoms and slow the kidney disease progression in patients with CKD (including DN), which might be another cause for the hypoalbuminemia. Ketoanalogs, which contained the essential amino acids, in conjunction with LPD treatment had been reported to delay the renal function decline, reduce albuminuria, and improve lipid metabolism without deteriorating the nutritional status—the underlying mechanism necessitating further research to clarify [42].

Some limitations in this study should be noted. First, it was a retrospective cohort study and selection bias was inevitable. Second, the association between serum albumin level changes and the renal prognosis should be cautiously regarded as correlation instead of causality. Third, the serum albumin levels were only analyzed at the baseline and the renal outcome was evaluated using an e-GFR. Fourth, oxidative modifications of serum albumin could also lead to “fictitious” hypoalbuminemia due to the underestimation of actual albumin levels using the BCG method in this study. Finally, we did not control the therapeutic interventions or dietary intake at baseline and during the follow-up.

## 5. Conclusions

In summary, our study found that the lower serum level of albumin was associated with the reduced kidney function and poor renal prognosis in patients with T2DM and DN, independent of clinical and histopathological features. Further, more clinical researches are warranted to verify whether the correction of hypoalbuminemia could improve renal prognosis in patients with DN.

## Figures and Tables

**Table 1:** Baseline clinical and pathological findings in groups stratified according to the serum albumin level.

Variables	Normal group ≥ 35 g/L (*n* = 87)	Mild group 30-35 g/L (*n* = 34)	Moderate group 25-30 g/L (*n* = 36)	Severe group < 25 g/L (*n* = 31)	*p* value
Clinical findings					
Age (year)	52.45 ± 9.66	53.41 ± 8.93	53.06 ± 6.32	52.26 ± 8.88	0.935
Gender (male)	60 (69.0%)	25 (73.5%)	22 (61.1%)	22 (71.0%)	0.704
Duration of diabetes (months)	84 (24-132)	48 (24-120)	120 (36-156)	120 (84-132)	0.054
DR (%)	34 (39.1%)	20 (58.8%)	19 (52.8%)	22 (71%)^†^	0.013
Cigarette smoking (%)	40 (46.0%)	19 (55.9%)	18 (50.0%)	16 (51.6%)	0.790
SBP (mmHg)	144.26 ± 19.80	149.91 ± 30.06	148.86 ± 24.25	153.71 ± 21.87	0.224
DBP (mmHg)	86.01 ± 12.43	84.59 ± 11.85	84.60 ± 12.49	89.32 ± 10.80	0.354
Hypertension (%)	77 (88.5%)	29 (85.3%)	32 (88.9%)	30 (96.8%)	0.391
Hematuria (%)	39 (44.8%)	24 (72.7%)^†^	27 (79.4%)^†^	22 (71.0%)	0.001
Initial proteinuria (g/d)	2.18 (0.93-3.66)	5.02 (3.29-7.81)^†^	5.55 (4.23-8.82)^†^	8.4 (6.15-14.42)^†‡^	<0.001
Nephrotic-range proteinuria (>3.5 g/d (%))	22 (25.3%)	21 (63.6%)^†^	30 (83.3%)^†^	29 (93.5%)^†‡^	<0.001
e-GFR (mL/min/1.73 m^2^)	65.22 (40.88-91.11)	52.14 (39.35-78.30)	42.16 (30.15-67.95)^†^	43.14 (28.92-59.22)^†^	<0.001
Stage 1,2,3a,3b,4,5 CKD (KDIGO)	24/24/10/21/8/0	5/8/10/6/5/0	2/9/3/13/9/0	0/7/7/9/8/0	—
Serum creatinine (mg/dL)	1.21 (0.85-1.70)	1.44 (1.07-1.67)	1.62 (1.19-2.31)^†^	1.80 (1.26-2.40)^†^	0.001
Uric acid (mmol/L)	413.60 ± 89.99	369.54 ± 71.93^†^	385.40 ± 67.20	364.94 ± 74.24^†^	0.006
FBS (mmol/L)	7.38 (5.86-9.10)	7.23 (5.88-9.75)	7.39 (4.74-10.22)	5.73 (4.64-8.59)	0.451
HbA1c (%)	6.90 (6.10-8.40)	7.55 (6.43-8.45)	7.30 (6.30-8.70)	6.80 (5.95-8.40)	0.706
Triglyceride (mmol/L)	1.83 (1.29-2.39)	1.63 (1.21-2.32)	1.63 (1.03-2.46)	1.82 (1.26-2.59)	0.557
Total cholesterol (mmol/L)	4.77 ± 1.20	5.03 ± 1.18	6.28 ± 1.82^†‡^	6.48 ± 2.03^†‡^	<0.001
Hemoglobin (g/L)	129.45 ± 26.25	116.91 ± 27.55	107.46 ± 24.64^†^	103.53 ± 19.16^†^	<0.001
Progressed to ESRD (%)	12 (13.8%)	13 (38.2%)	27 (75%)	24 (77.4%)	—
Histopathological findings					
Glomerular class					<0.001
I	10 (11.5)	0 (0)	0 (0)	0 (0)	
IIa	29 (33.3)	1 (2.9)	1 (2.8)	3 (9.7)	
IIb	8 (9.2)	6 (17.6)	5 (13.9)	1 (3.2)	
III	27 (31)	20 (58.8)	26 (72.2)	22 (71)	
IV	13 (14.9)	7 (20.6)	4 (11.1)	5 (16.1)	
IFTA					0.014
0	5 (5.7)	0 (0)	1 (2.8)	0 (0)	
1	49 (56.3)	15 (44.1)	14 (38.9)	8 (25.8)	
2	24 (27.6)	17 (50)	20 (55.6)	19 (61.3)	
3	9 (10.3)	2 (5.9)	1 (2.8)	4 (12.9)	
Interstitial inflammation					0.001
0	10 (11.5)	1 (2.9)	1 (2.8)	0 (0)	
1	70 (80.5)	26 (76.5)	21 (58.3)	21 (67.7)	
2	7 (8)	7 (20.6)	14 (38.9)	10 (32.3)	
Arteriolar hyalinosis					0.006
0	20 (23)	0 (0)	2 (5.6)	4 (12.9)	
1	41 (47.1)	14 (41.2)	16 (44.4)	15 (48.4)	
2	26 (29.9)	20 (58.8)	18 (50)	12 (38.7)	
Therapy					
RAAS inhibitor (%)	70 (80.5)	28 (82.4)	29 (80.6)	25 (80.6)	0.996
Oral hypoglycemic agents (%)	45 (51.7)	15 (44.1)	5 (13.9)^†‡^	12 (38.7)	0.002
Insulin therapy (%)	59 (67.8)	26 (76.5)	29 (82.9)	24 (77.4)	0.330

Abbreviations: DR: diabetic retinopathy; SBP: systolic blood pressure; DBP: diastolic blood pressure; e-GFR: estimated glomerular filtration rate; FBS: fasting blood sugar; HbA1c: glycosylated hemoglobin; ESRD: end-stage renal disease; RAAS: renin-angiotensin-aldosterone system. ^†^ *p* < 0.05 versus the normal group. ^‡^ *p* < 0.05 versus the mild group.

**Table 2:** Correlations between the albumin level and histopathological and clinical findings.

	Variables	Correlation coefficient (*r*)	*p* value
Albumin	Glomerular class	-0.394	<0.001^∗^
	IFTA	-0.269	<0.001^∗^
	Interstitial inflammation	-0.378	<0.001^∗^
	Arteriolar hyalinosis	-0.219	0.003^∗^
	Log e-GFR (mL/min/1.73 m^2^)	0.334	<0.001^a^
	Log proteinuria (g/d)	-0.661	<0.001^b^
	Hemoglobin (g/L)	0.325	<0.001^b^
	Uric acid (mmol/L)	0.331	<0.001^b^
	Total cholesterol (mmol/L)	-0.424	<0.001^b^

IFTA: interstitial fibrosis and tubular atrophy. ^∗^Spearman's correlation analysis. A two-tailed *p* < 0.05 was considered statistically significant. ^a^Partial correlation analysis for adjusting the baseline age and gender. ^b^Partial correlation analysis for adjusting the baseline age, gender, and log e-GFR.

**Table 3:** Associations between serum albumin and renal outcomes.

		Hazard ratios (95% confidence interval) & *p* value			
	Serum albumin, median (range) (g/L)	Unadjusted	Model 1^a^	Model 2^b^	Model 3^c^
Per 1 SD serum albumin	34.15 (14.00-48.80)	0.35 (0.27-0.46) *p* < 0.001	0.18 (0.06-0.58) *p* = 0.004	0.20 (0.06-0.66) *p* = 0.008	0.21 (0.06-0.67) *p* = 0.009
Normal group	40.20 (35.10-48.80)	Reference	Reference	Reference	Reference
Mild group (35-30 g/L)	33.25 (30.30-34.90)	2.99 (1.36-6.61) *p* = 0.007	1.45 (0.49-4.31) *p* = 0.501	2.24 (0.72-6.90) *p* = 0.162	2.09 (0.67-6.56) *p* = 0.205
Moderate group (25-30 g/L)	27.50 (25.20-29.90)	6.03 (3.05-11.95) *p* < 0.001	4.67 (1.59-13.74) *p* = 0.005	6.56 (2.05-20.94) *p* = 0.001	6.20 (1.95-19.76) *p* = 0.002
Severe group (<25 g/L)	22.00 (14.00-24.80)	13.74 (6.63-28.44) *p* < 0.001	6.14 (1.27-29.66) *p* = 0.024	10.61 (1.87-60.07) *p* = 0.008	7.37 (1.24-43.83) *p* = 0.028

Serum albumin was analyzed as a continuous variable with hazard ratios (HRs) calculated per SD increment. SD: standard deviation. ^a^Model 1 adjusted for the baseline age, gender, duration of diabetes, DR (yes or no), hypertension (yes or no), hematuria, total cholesterol, hemoglobin, and log proteinuria and e-GFR. ^b^Model 2 adjusted for covariates in model 1 plus renal pathological findings (the glomerular class, IFTA, interstitial inflammation scores, and arteriolar hyalinosis). ^c^Model 3 adjusted for covariates in model 2 plus RAAS inhibitor use (yes or no).

## Data Availability

The clinical and pathological data of all the DN patients used to support the findings of this study have not been made available to protect the patients' rights of privacy.

## Supplementary Materials

Supplementary Materials Supplementary Table 1: the clinical features of the excluded and included patients in this study. Supplementary Table 2: Cox regression analysis of risk factors for renal outcomes in DN patients.
