# Supplementary material for: The Level of Serum Albumin Is Associated with Renal Prognosis in Patients with Diabetic Nephropathy
Source: J Diabetes Res. 2019 Feb 17;2019:7825804. doi: 10.1155/2019/7825804 (PMC6398001; doi:10.1155/2019/7825804)
Supplement: Supplementary Materials — Supplementary Table 1: the clinical features of the excluded and included patients in this study. Supplementary Table 2: Cox regression analysis of risk factors for renal outcomes in DN patients. [file 7825804.f1.docx]

**Supplement table 1.** The clinical features of the excluded and included patients in this study.

| **Variables** | **Excluded patients (n=61)*** | **Included patients (n=188)** | **P value** |
| --- | --- | --- | --- |
| **Age (year)** | 51.12±9.43 | 52.71±8.79 | 0.253 |
| **Gender (Male)** | 40(65.6) | 129(68.6) | 0.658 |
| **Duration of diabetes (Months)** | 72(36-120) | 90(36-132) | 0.367 |
| **DR (%)** | 27(44.3) | 95(50.5) | 0.395 |
| **BMI(kg/m^2^)** | 25.03±3.58 | 25.88±4.24 | 0.374 |
| **SBP (mm Hg)** | 142.80±22.01 | 147.72±23.21 | 0.150 |
| **DBP (mm Hg)** | 84.95±14.91 | 86.04±12.09 | 0.568 |
| **Hypertension (%)** | 50(82.0) | 168(89.4) | 0.129 |
| **Hematuria (%)** | 38(64.4) | 112(60.5) | 0.595 |
| **Initial proteinuria (g/d)** | 4.92(2.82-8.34) | 4.09(1.88-6.75) | 0.069 |
| **Uric acid (mmol/L)** | 367(318-421) | 382.8(339.25-434) | 0.112 |
| **FBS (mmol/L)** | 7.54(5.46-10.38) | 7.13(5.45-9.34) | 0.703 |
| **HbA1c (%)** | 7.25(5.98-8.08) | 7.10(6.20-8.40) | 0.743 |
| **Triglyceride(mmol/L)** | 1.66(1.24-2.51) | 1.74(1.21-2.42) | 0.847 |
| **Total cholesterol(mmol/L)** | 5.06(4.26-6.36) | 5.18(4.35-6.25) | 0.671 |
| **Hemoglobin (g/L)** | 117(100.5-145) | 117(100-137) | 0.671 |
| **Serum albumin(g/L)** | 31.25(25.20-39.43) | 34.15(27.28-39.38) | 0.158 |

*The excluded 61 patients included 57 patients with follow-up time <1year and 4 patients without data of albumin.

**Supplementary -table 2. Cox regression analysis of risk factors for renal outcomes in DN patients.**

| **Variables** | **Unadjusted** | | | **Model 3** | | |
| --- | --- | --- | --- | --- | --- | --- |
|  | **HR** | **95%CI** | **P value** | **HR** | **95%CI** | **P value** |
| **Age(years)** | **1.002** | **0.977-** **1.028** | **0.849** | **0.990** | **0.954-1.027** | **0.583** |
| **Gender (male)** | **0.847** | **0.518-** **1.384** | **0.507** | **1.274** | **0.578-2.809** | **0.548** |
| **Duration of diabetes(months)** | **1.003** | **1.000-** **1.006** | **0.077** | **1.001** | **0.997-1.005** | **0.603** |
| **Diabetic retinopathy (yes *vs* no)** | **1.926** | **1.211-** **3.062** | **0.006** | **1.359** | **0.750-2.465** | **0.312** |
| **Cigarette smoking (yes *vs* no)** | **1.11** | **0.707-1.741** | **0.651** | **-** | **-** | **-** |
| **Hypertension (yes *vs* no)** | **1.295** | **0.618-** **2.714** | **0.493** | **0.957** | **0.375-2.443** | **0.927** |
| **Hematuria (%)** | **1.783** | **1.071-2.970** | **0.026** | **0.934** | **0.498-1.752** | **0.831** |
| **Uric acid (mmol/L)** | **0.998** | **0.995-1.001** | **0.119** | **-** | **-** | **-** |
| **HbA1c (%)** | **0.929** | **0.820-** **1.051** | **0.242** | **-** | **-** | **-** |
| **Total cholesterol(mmol/L)** | **1.168** | **1.030-1.326** | **0.016** | **0.558** | **0.297-1.051** | **0.071** |
| **Hemoglobin (g/L)** | **0.967** | **0.958-0.977** | **<0.001** | **0.999** | **0.981-1.016** | **0.891** |
| **Log-Proteinuria** | **11.773** | **5.565-** **24.909** | **<0.001** | **2.917** | **0.883-9.637** | **0.079** |
| **e-GFR(ml/min/1.73m^2^)** | **0.965** | **0.954-0.976** | **<0.001** | **0.964** | **0.945-0.983** | **<0.001** |
| **Glomerular class** | **1.934** | **1.487-2.515** | **<0.001** | **1.513** | **0.948-2.414** | **0.083** |
| **IFTA** | **1.507** | **1.106-2.054** | **0.009** | **1.079** | **0.298-3.902** | **0.908** |
| **Interstitial inflammation** | **2.653** | **1.672-4.209** | **<0.001** | **3.64** | **0.588-22.513** | **0.165** |
| **Arteriolar hyalinosis** | **1.356** | **0.971-1.893** | **0.074** | **0.769** | **0.497-1.189** | **0.238** |
| **RAAS inhibitor (yes *vs* no)** | **0.948** | **0.546-1.647** | **0.851** | **1.795** | **0.854-3.772** | **0.123** |
| **Per 1 SD serum albumin** | **0.351** | **0.268- 0.459** | **<0.001** | **0.205** | **0.063-0.670** | **0.009** |
